# CDC Support for Global Public Health Emergency Management

**DOI:** 10.3201/eid2313.170542

**Published:** 2017-12

**Authors:** Daniel J. Brencic, Meredith Pinto, Adrienne Gill, Michael H. Kinzer, Luis Hernandez, Omer G. Pasi

**Affiliations:** US Centers for Disease Control and Prevention, Atlanta, Georgia, USA (D.J. Brencic, M. Pinto, A. Gill, L. Hernandez);; US Centers for Disease Control and Prevention, Dakar, Senegal (M.H. Kinzer);; US Centers for Disease Control and Prevention, Yaoundé, Cameroon (O.G. Pasi)

**Keywords:** Public health, global health, emergencies, emergency operations centers, International Health Regulations, global health security, IHR 2005

## Abstract

Recent pandemics and rapidly spreading outbreaks of infectious diseases have illustrated the interconnectedness of the world and the importance of improving the international community’s ability to effectively respond. The Centers for Disease Control and Prevention (CDC), building on a strong foundation of lessons learned through previous emergencies, international recognition, and human and technical expertise, has aspired to support nations around the world to strengthen their public health emergency management (PHEM) capacity. PHEM principles streamline coordination and collaboration in responding to infectious disease outbreaks, which align with the core capacities outlined in the International Health Regulations 2005. CDC supports PHEM by providing in-country technical assistance, aiding the development of plans and procedures, and providing fellowship opportunities for public health emergency managers. To this end, CDC partners with US agencies, international partners, and multilateral organizations to support nations around the world to reduce illness and death from outbreaks of infectious diseases.

Recent public health events, such as the 2016 Zika outbreak and 2009 influenza A(H1N1) pandemic, have illustrated the interconnectedness of the world and the importance of global health security. Outbreaks of new and highly infectious diseases that start in remote parts of the world can quickly spread to large, urban populations. When Ebola virus disease appeared in Nigeria in 2014, what could have been an explosion of cases was quickly contained, in part because of prior emergency management investment by the government of Nigeria, with assistance from the US Centers for Disease Control and Prevention (CDC) and other organizations. Nigeria’s ability to use public health emergency management (PHEM) principles to rapidly detect and respond proved invaluable in quickly and effectively stopping the spread of Ebola throughout the country and illustrates the effect of a strong PHEM program (*1*,*2*).

In 2004, in response to the changing landscape of public health emergencies, the World Health Organization (WHO) led an effort, with support from CDC and other international organizations, to update the International Health Regulations (IHR), leading to adoption of the IHR 2005 (*3*) (Figure 1). According to WHO, “One of the most important provisions in the IHR is the obligation for all States Parties to establish core capacities to detect, assess, notify and report events, and to respond to public health risks and emergencies” (*4*). All member countries had until 2012 to conduct self-assessments and report their progress to WHO. In 2014, WHO, CDC, and other partners launched the Global Health Security Agenda (GHSA) to further advance national capacities to rapidly detect, respond to, and control public health emergencies and thereby comply with IHR 2005 (*5*). Although many countries were able to manage small outbreaks within their borders, the introduction of new diseases and the increased spread of disease from international travel exposed the need for a more purposeful and streamlined approach to manage these public health emergencies.

**Figure 1:**
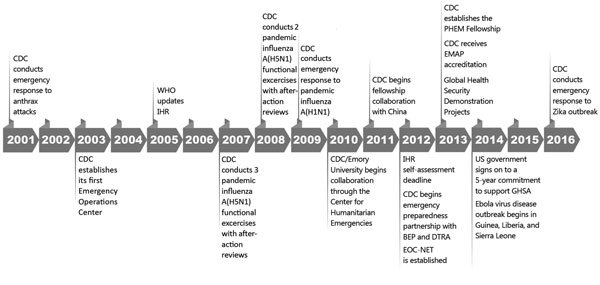
Timeline of CDC's support for development of global public health emergency management, 2001–2016. BEP, Biosecurity Engagement Program; CDC, Centers for Disease Control and Prevention; DTRA, Defense Threat Reduction Agency, US Department of Defense; EMAP, Emergency Management Accreditation Program; EOC-NET, Public Health Emergency Operations Centre Network; GHSA, Global Health Security Agenda; IHR, International Health Regulations; PHEM, public health emergency management; WHO, World Health Organization.

In the same timeframe that IHR 2005 was being written, CDC began to build its own preparedness and response program as a direct result of the increasing risk for public health threats and increased terrorism around the world (*6*). Using foundational emergency management principles, including the Incident Management System (IMS), CDC established its first permanent Emergency Operations Center (EOC) in 2003 and activated it soon after for the agency’s response to the 2003 outbreak of severe acute respiratory syndrome (*7*). Since then, CDC has aimed to strengthen its emergency management program through exercises and responses to meet industry emergency response standards and, in 2013, became the first federal agency to receive full accreditation from the Emergency Management Accreditation Program (*8*). Building on a strong foundation of lessons learned through previous emergencies, national accreditation, international recognition, and technical expertise, CDC has established itself as a world leader in PHEM and begun to help other entities strengthen their capacity. CDC, as outlined in its Global Health Strategy (2012–2015), now collaborates “with host country governments and partner organizations to strengthen health security by improving the ability of countries to prepare for and respond to disease threats on a global scale” (*9*).

## History of CDC’s Global PHEM Work

CDC’s global footprint has grown considerably during the past 2 decades. As of 2016, CDC has 342 staff stationed in ≈50 countries and ≈40 staff detailed to international organizations and is supported by ≈1,368 locally employed staff from host countries (*10*). Starting in 2009, CDC hired local emergency coordinators in Guatemala, Kenya, Egypt, Kazakhstan, Thailand, and China. As CDC’s EOC became increasingly involved in managing public health responses, and the role of the emergency coordinators evolved, CDC began to focus on assisting host country ministries of health with institutionalizing emergency preparedness and response activities. The objectives were to train on IMS and risk communication, complete public health capacity assessments, develop emergency preparedness plans, conduct tabletop exercises, and advise about EOC facility development. Through these efforts, CDC laid the foundation for further technical assistance.

The CDC emergency coordinators have been a valuable asset in this endeavor by providing technical knowledge in emergency preparedness and cultural understanding of the local contexts. In particular, during 2011–2015, the emergency coordinator based at the CDC Central America Regional Office supported the Risk Management Departments of 8 ministries of health in the Central American Region through the Council of Ministries of Health of Central America cooperative agreement. The development of public health emergency response plans and the development of EOCs led to ≈3,800 hours of training to ≈400 staff from 9 countries in Central and South America. As a result of these collaborations with CDC, Central America is better prepared to manage public health emergencies.

## Fellowships and Delegations

CDC’s growing role providing PHEM technical assistance coincided with countries’ self-assessments for the 2012 deadline to report on progress toward achieving core capacities outlined in IHR 2005. During this time, requests increased to CDC for PHEM technical assistance, and CDC began to provide short-term, in-country emergency preparedness trainings and to host international delegations at the CDC EOC. During 2008–2011, the number of in-country delegations visiting CDC and learning about the US national-level PHEM program increased by 41%. CDC continues to host delegations and collaborates with local partners in Atlanta to enable visitors to observe PHEM at federal, state, and local levels.

Because of the benefits gained through the visits to CDC, several countries expressed interest in comprehensive fellowship opportunities to learn how CDC manages public health emergencies. In 2011, through a cooperative agreement between CDC, the Chinese Center for Disease Control and Prevention, and the National Health and Family Planning Commission of the People’s Republic of China, CDC hosted fellows from China for a year-long study tour. As Chinese institutions became more advanced in their plans and training, they sent staff for shorter fellowships to be embedded with CDC emergency management teams and receive specialized training in the areas of emergency plan development, EOC management and operations, and exercises and evaluation.

The interest of sending international public health staff to CDC to learn about public health emergency preparedness and response continued to grow, and in 2013 CDC established the Public Health Emergency Management Fellowship (PHEMF) program in Atlanta to build PHEM capacity among members of the international public health community through residential training and mentorship. Fellows complete a comprehensive, standardized study program in core emergency management functions that includes operations, planning, risk communications, and logistics. They observe CDC EOC responses and conduct site visits to improve their familiarity with PHEM in the field. The program enables fellows to interact with, and learn from, stakeholders of CDC’s emergency management system, including federal, state, and local partners.

The PHEMF curriculum is guided by a global PHEM Core Competency Model, currently in development, which encompasses 7 competencies: leadership, emergency management frameworks, emergency management functions, emergency management communication, partnership and collaboration, training development and facilitation, and evaluation. With mentorship from CDC subject matter experts, fellows apply their learning to develop a personalized toolkit of products to be used by their ministries of health. Specific products in the toolkits may include standard operating procedures (SOPs), draft all-hazards or hazard-specific plans, or Web-based systems for EOC messaging.

By December 2016, CDC had trained 39 fellows from 25 countries. As leaders within their respective organizations, returning fellows facilitate the expansion of PHEM within their countries and have assumed key roles as leaders and managers of emergency response units in Africa, Asia, and the Middle East.

### Building a Community of Practice

In the years leading up to the 2012 self-assessment on IHR 2005 capacities, worldwide need increased for guidelines and standards for building PHEM capacity. In fact, by June 2012, only “42 of 193 [21.76%] States Parties declared that they had met their core capacity requirements” (*4*), and most countries had requested a 2-year extension. To fill this need, CDC and other international organizations focused their global technical assistance on countries’ IHR 2005 requirements. However, after 2 years of such support, in 2014, WHO reported that “at the time of…[the] second Review Committee meeting, 64 States Parties [33.16%] had indicated that they met the minimum core capacity standards” (*4*), which indicated that additional effort was needed.

Ongoing reviews of countries’ PHEM capabilities demonstrated a lack of clear international guidelines for program implementation. Therefore, WHO, with support from CDC, the Chinese Center for Disease Control and Prevention, the European Centre for Disease Prevention and Control, and others, established the Public Health Emergency Operations Centre Network (EOC-NET), which strives to identify best practices in PHEM and promote EOC capacity-building activities in member states (*11*). As a member of EOC-NET, in 2013 CDC supported WHO’s systematic review of EOCs and technical consultations through 4 working groups that aimed to develop guidance and standards for building, maintaining, and managing Public Health EOCs (PHEOCs).

The direct result of the EOC-NET work was publication of the Framework for a Public Health Emergency Operations Centre in 2015 as a first step in creating internationally recognized minimum common standards for PHEOCs. The Framework “outlines the key concepts and essential requirements for developing and managing… a PHEOC in order to achieve a goal-oriented response to public health emergencies and unity of effort among response agencies” (*12*). These guidelines provide a framework for public health emergency managers and practitioners to build the core capacity elements necessary for effective responses to public health emergencies.

### Initiatives in Global Public Health Emergency Management

In 2013, CDC partnered with the ministries of health in Uganda and Vietnam as part of the Global Health Security Demonstration Project to show what public health capacity could be developed in 6 months with a concentrated commitment of technical assistance and resources. Although each country faced unique hazards and challenges, both projects focused on 3 main areas: 1) strengthening laboratory systems, 2) improving information gathering and sharing, and 3) developing a highly functioning PHEOC (*13*,*14*). CDC worked with the countries to develop SOPs and provide emergency management training for their PHEOC staff. At the culmination of the project, each country underwent a series of drills to demonstrate its increased capacity in the 3 target areas and showed significant improvements.

In 2014, the US government signed on to a 5-year commitment to support GHSA, an international collaboration among partner nations and international organizations intended to serve as a roadmap for countries to reach the capacities outlined in IHR 2005 (*5*). The goal of GHSA is to prepare nations around the world to more quickly and effectively detect and respond to infectious disease threats to reduce morbidity and mortality and prevent the global spread of disease (*15*). Eleven GHSA Action Packages are organized around Prevent, Detect, and Respond (*16*), and CDC’s strong foundation in PHEM has positioned the agency to provide effective technical assistance for the Respond 1 Action Package: Emergency Operations Centers.

**Figure 2:**
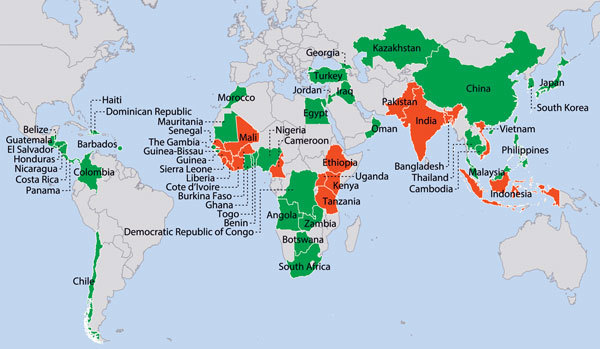
Centers for Disease Control and Prevention public health emergency management (PHEM) engagements, 2008–2016. Red indicates Global Health Security Agenda PHEM engagement; green indicates other PHEM engagement; gray indicates no PHEM engagement.

The Respond 1 goal is for a country to have “a PHEOC functioning according to minimum common standards and trained EOC staff capable of activating a coordinated emergency response within 120 minutes of the identification of a public health emergency” (*16*). Although the Action Package goal highlights the need for each country to have a functioning PHEOC, what the Global Health Security Demonstration Project showed was that a fully coordinated response can be accomplished only through a comprehensive PHEM program. Through GHSA, CDC provides technical assistance to 17 countries in 3 areas: training and mentoring of PHEM staff; reinforcing sufficient PHEOC infrastructure; and developing streamlined systems, including plans, SOPs, and connections with other ministries of health (Figure 2).

At the same time CDC began to support GHSA, the Ebola virus disease outbreak struck West Africa. Using the lessons learned in the Global Health Security Demonstration Project, in September 2014, CDC provided emergency management technical assistance to develop PHEOCs in Guinea, Liberia, and Sierra Leone and the surrounding countries (*17*). The Ebola outbreak substantially weakened the already limited public health systems in the 3 affected countries. CDC’s emergency management assistance focused on developing IMS goals and objectives, coordinating infrastructure improvements for increased collaboration, assisting with logistics, and training staff on PHEM principles. The progress in these 3 countries demonstrated that “rudimentary emergency management capacities can be rapidly established in countries with the application of focused technical assistance” (*17*). The response to this outbreak provided CDC with a unique opportunity to understand and overcome the challenges of providing technical assistance to countries with limited emergency management capacity, which would inform the approach for GHSA.

Throughout 2015, US government interagency delegations composed of officials from the Department of State (DOS), US Agency for International Development (USAID), Department of Defense (DOD), and CDC participated in GHSA launch meetings in 17 countries. As part of the implementation of the GHSA, countries developed 1-year work plans based on 5-year roadmaps that outlined a country’s priorities, objectives, and activities and how the US government, other foreign governments, and international organizations would provide financial support and technical assistance. CDC developed a standardized package of technical assistance activities using a variety of foundational emergency management documents, including the International Organization for Standardization 22300 Societal Security series (*18*–*20*); the WHO EOC Framework (*10*); and industry-specific standards, such as the National Fire Protection Association 1600 Standard on Disaster/Emergency Management and Business Continuity/Continuity of Operations (*21*). CDC then worked with ministries of health to customize work plans based on the country’s baseline capacity and 5-year strategic goals for a PHEOC.

Through collaboration with in-country partners, CDC assists countries with public health threat and hazard identification and risk assessments; design of PHEOC policies, protocols, and guidelines; strategic and operational plans; planning for the physical design of a PHEOC; training PHEOC staff; and developing and executing exercises to validate activities. The effect of this work has been demonstrated in multiple ways. For example, in Cameroon, 33 Ministry of Health staff received basic PHEM training and participated in a follow-up exercise, and 26 participated in a workshop to develop and validate 11 priority SOPs for the PHEOC. A PHEMF graduate served as the incident manager for an influenza A(H5N1) outbreak and applied newly acquired skills in IMS to coordinate and manage the Ministry of Health’s emergency response (*22*). In 2016 alone, Cameroon has seen a decrease in the time it takes to activate the PHEOC from 8 weeks (cholera outbreak) to 1 week (Lassa fever outbreak) to 24 hours (H5N1 outbreak), and coordination between animal and human health stakeholders has substantially improved.

Senegal needed a PHEM program early in the West Africa Ebola outbreak when an Ebola-positive person traveled from Guinea to the capital, Dakar. Since that time, emergency management has improved substantially through development of a PHEOC with support from CDC, the DOD’s Defense Threat Reduction Agency (DTRA), and USAID. The Ministry of Health has trained permanent PHEOC staff, developed plans and procedures, and participated in 2 simulation exercises. The PHEOC assets also have been linked to national systems in public health surveillance, laboratory, human resources, and other sectors through joint strategic planning and simulation exercises. Both Cameroon and Senegal are emerging as regional PHEM leaders and are leading initiatives to share resources and exchange lessons learned from emergency responses with other West Africa countries. Of the 17 GHSA countries, 16 have received in-country CDC technical assistance and completed data collection for planning emergency management technical assistance, and 12 have held in-country CDC emergency management trainings.

## Leveraging Partnerships to Increase PHEM Impact

Emergency management principles strive to streamline coordination and collaboration. Therefore, CDC works with US agencies and international partners to reach the goals of IHR 2005. CDC coordinates with DOS, DOD, USAID, and others to leverage resources and partnerships to expand emergency management technical assistance to countries. For example, since 2012, CDC has partnered with the DOS Biosecurity Engagement Program (BEP) to support biosecurity-related emergency preparedness. In India, CDC collaborated with the National Centre for Disease Control to develop a national-level PHEOC and is further strengthening emergency management capacity by developing PHEOCs at the state and district levels in Tamil Nadu. This network of PHEOCs will help India be better prepared to respond to biosecurity/biosafety threats. In Jordan, CDC partners with DTRA and DOS in 2 separate initiatives; through BEP, CDC has been working with the Ministry of Health Crisis Management Unit to develop a national-level PHEOC and provide training for Ministry of Health staff on the principles of emergency management. In addition, CDC has partnered with DTRA to bring different entities of the government of Jordan together for emergency preparedness planning, EOC training, and exercises focusing on civil–military coordination during humanitarian crises and health emergencies. These activities can streamline emergency management across Jordan’s government.

CDC’s partnerships and technical assistance also extend to large multilateral organizations and entities. CDC participates in WHO-led initiatives as subject matter experts in Joint External Evaluations, which assess a country’s capacity to prevent, detect, and respond to public health threats (*23*), and partners with WHO to conduct GHSA activities. CDC also provides the African Union with emergency management training and technical assistance in developing a continent-level PHEOC. CDC partners with other nations’ public health organizations, such as Public Health England and Public Health Agency of Canada, to leverage technical and language expertise and has joined with Emory University (Atlanta, GA, USA) through its Rollins School of Public Health Center for Humanitarian Emergencies to help develop the next generation of public health practitioners in humanitarian emergencies, emergency preparedness, and response (*24*).

## Public Health Impact

Countless examples throughout the past few years have shown that diseases know no borders and can rapidly spread across land and sea. Increasing the international community’s ability to rapidly and effectively respond to public health threats ensures the broader global health security of all people. In resource-limited environments, emergency response is centered on achieving the biggest public health impact. PHEM components, like preparedness plans, SOPs, and EOCs, contribute to faster and more efficient responses during emergencies which enable a greater reduction in morbidity and mortality.

## Limitations

Successes have occurred in capacity development in some countries; however, challenges and limitations remain to building PHEM capacity around the globe. Although the WHO EOC Framework provides guidelines for countries on how to build a PHEM program, each country faces unique circumstances and challenges in implementing these programs. Laws, policies, and authorities vary substantially, and because PHEM is still a relatively new concept for most developing countries, high-level support must be cultivated. Countries with limited financial and human resources must prioritize planning for the most immediate and dire threats, and preparedness planning can seem an unaffordable luxury of time and resources. CDC and other international partners have provided technical assistance and resources, but transitioning to managing public health emergencies through a PHEOC still requires major commitment and input from a ministry of health to progress from having functionality to being fully operational. It is often expensive for countries to dedicate staff to work only on PHEM without drawing resources from other parts of the ministry of health. Efforts to strengthen PHEM capacity must build on existing emergency response structures. Any augmentation of technology and infrastructure also should improve nonemergency capability to be sustainable and effective.

## Conclusions and Next Steps

The ability to detect and respond locally to public health threats has been needed for generations. However, as the world becomes more interconnected, countries are realizing an increased need to prepare for public health threats from across the globe. Furthermore, global health security relies on the capacity of all countries to comply with IHR 2005 and rapidly detect, respond to, and control public health emergencies. This realization has increased the demand for PHEM technical assistance in building countries’ sustainable capacity. Although CDC has focused on providing PHEM technical assistance to a select number of countries through programs such as the GHSA and partners such as BEP and DTRA, the need for PHEM assistance exceeds current support. The Zika outbreak in Central and South America is just one example of how nations outside these programs are susceptible to threats previously limited only to countries on other continents.

Providing technical assistance to countries during an outbreak or public health emergency is important; however, CDC encourages countries to invest in preparing for emergencies by highlighting the effect of an effective PHEM program on a response. CDC is focusing on regional workshops to bring together neighboring countries for trainings to increase communication and collaboration to leverage expertise across nations with similar threats and hazards. Continued close collaboration and partnership across the US government and with international organizations will enable more to be accomplished through leveraging individual institutional strengths. CDC aims to standardize the PHEM approach to respond to public health emergencies by continuing to assist WHO, through initiatives such as EOC-NET, to create global standards for PHEM. With this effort, CDC aims to reach its goal of saving lives and protecting people while making the world a safer place from disease outbreaks and other public health threats.
